# Long persistence of severe acute respiratory syndrome coronavirus 2 swab positivity in a drowned corpse: a case report

**DOI:** 10.1186/s13256-022-03297-8

**Published:** 2022-02-09

**Authors:** Martina Bonelli, Enrica Rosato, Marcello Locatelli, Angela Tartaglia, Pietro Falco, Claudia Petrarca, Francesca Potenza, Verena Damiani, Domitilla Mandatori, Vincenzo De Laurenzi, Liborio Stuppia, Cristian D’Ovidio

**Affiliations:** 1 Section of Legal Medicine, Center for Advanced Studies and Technology (CAST), University “G. d’Annunzio” of Chieti-Pescara, 66100 Chieti, Italy; 2 Department of Pharmacy, University of Chieti-Pescara “G. d’Annunzio”, Via dei Vestini 31, 66100 Chieti, Italy; 3 Division of Legal Medicine, SS. Annunziata Hospital, Chieti, Italy; 4 Allergy and Immunotoxicology and Occupational Biorepository, Center for Advanced Studies and Technology (CAST), University “G. d’Annunzio” of Chieti-Pescara, 66100 Chieti, Italy; 5 Center for Advanced Studies and Technology (CAST), University “G. d’Annunzio” of Chieti-Pescara, 66100 Chieti, Italy

**Keywords:** SARS-CoV-2, COVID-19, Postmortem swab, Postmortem positivity, COVID-19 on drowned cadaver

## Abstract

**Background:**

Since the beginning of the worldwide spread of severe acute respiratory syndrome coronavirus 2 to date, important knowledge has been obtained about the virus behavior in living subjects and on inanimate surfaces; however, there is still a lack of data on virus persistency on dead bodies and the risk of contagion from cadavers.

**Case presentation:**

The present case shows the persistency of the severe acute respiratory syndrome coronavirus 2 viral genome in nasopharyngeal swabs performed on a drowned Caucasian man, aged 41 years old, who was completely asymptomatic when he was alive, up to 41 days after death. Specific real-time reverse transcriptase-polymerase chain reaction (TaqMan 2019-nCoV Assay Kit v2; Thermo Fisher Scientific, Italy and Realquality RQ-SARS-CoV-2, AB Analytical) was used to evaluate the swabs.

**Conclusions:**

This data reflect the importance of postmortem swabs in all autopsy cases, and not only in potential severe acute respiratory syndrome coronavirus 2-related death, and also highlight the necessity to evaluate virus positivity a long time after the moment of death, even if a low initial viral load was assessed.

## Background

As reported by the World Health Organization, from the outbreak of the pandemic until 25 February 2021, a total of 112,209,815 confirmed cases of severe acute respiratory syndrome coronavirus 2 (SARS-CoV-2) have been recorded, including 2,490,776 deaths [1]. Worldwide statistical–epidemiological data on SARS-CoV-2 spread show a mortality rate ranging between 0.8% and 4.2% [2], most of these occurring among the elderly (age > 80 years), immunocompromised, or patients with comorbidities [3].

Through autopsies, pathologists can provide an important contribution to better understand the phenomenology of death in SARS-CoV-2-positive patients, and to date, there is no evidence about the possibility of being infected from a corpse.

## Case presentation

Herein we report the case of a 41-year-old Ukrainian man who went missing after swimming in the sea with a friend due to unfavorable weather conditions. About 16 hours after the event, the body was found wedged between rocks and an autopsy was ordered. Due to the current pandemic situation, having no information about the possible antemortem SARS-CoV-2 positivity of the man, who was described as completely asymptomatic, a postmortem nasopharyngeal swab was taken before the autopsy, according to the current guidelines. The swab identified SARS-CoV-2 positivity. Circumstantial data and external inspection of the corpse allowed death to be considered compatible with drowning.

Due to pending burial authorizations, the corpse was kept in the Chieti Hospital morgue, respecting the guidelines on management of SARS-CoV-2 deceased. The waiting period allowed us to follow the evolution of the positivity of the virus by performing multiple nasopharyngeal swabs.

### Collection phase

The whole observation period of the corpse was 41 days. During this period, the body was stored in a cold room at 4 °C, inside a sealed and disinfected waterproof bag, in compliance with the guidelines issued for the management of SARS-CoV-2 corpses [4].

Twenty-eight nasopharyngeal swabs were performed on the corpse. The collection of the samples was always performed by the same team, adequately prepared and with standardized procedures as per international guidelines [5] and established protocols [6, 7]. Since the collection of cadaver swabs does not induce any physiological reactions capable of generating aerosols, a negative-pressure room (BLS3) was not required. Two nasopharyngeal swabs (one for each nostril) were performed, using a kit consisting of two synthetic fiber swabs with plastic rod. After collection, each sample was placed inside a sterile tube containing 2–3 mL physiological solution with addition of an antibiotic. The tubes were then placed in two different 50-mL Falcon tubes, stored in a plastic bag with an adhesive closure, and then in another plastic bag with a zip closure. After sampling, the swabs were transported to the laboratory where they were stored for a maximum of 12 hours at a temperature of 4 °C, waiting to be processed.

### Laboratory phase

Specific real-time reverse transcriptase-polymerase chain reaction (RT-PCR) (TaqMan 2019-nCoV Assay Kit v2; Thermo Fisher Scientific, Italy) targeting RNA-dependent RNA polymerase was used to detect presence of SARS-CoV-2. This technique uses three genes: *ORF1ab*, *N gene*, and *S gene* to quantify the viral load by the number of cycles for the fluorescent signal to cross the threshold in RT-PCR. The threshold is 5000, the baseline is 5, and the cut-off is 37 cycles. A lower number of cycles means a higher viral load. According to the TaqMan 2019-nCoV Assay Kit v2, samples are considered “positive” when at least two genes have a cycle threshold value <37; if the cycle threshold value is “undetermined” or > 37 for two or three genes, then the sample is considered “negative.”

A second RT-PCR kit (Realquality RQ-SARS-CoV-2, AB Analytical) was also used to check the positivity of the swab and to introduce a cellular control. This kit analyzes S and *RdRP* viral genes and the *RnaseP* gene for cellular control.

## Results

All swabs performed during the observational period were reported as positive.

**Fig. 1:**
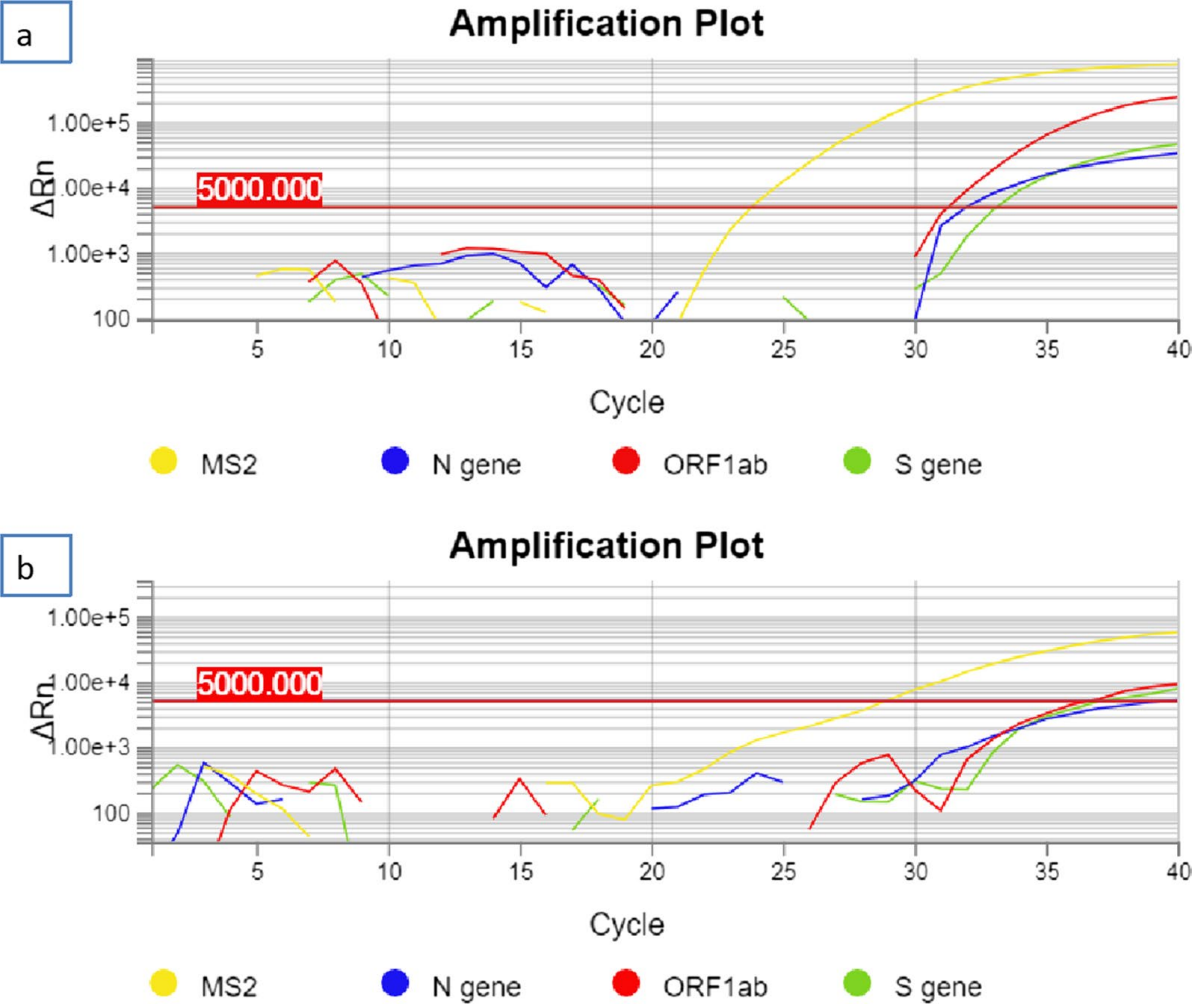
RT-PCR (TaqMan 2019-nCoV Assay Kit v2; Thermo Fisher Scientific, Italy). Amplification plot of first swab (**a**) and last swab (**b**)

We compared the results of RT-PCR (TaqMan 2019-nCoV Assay Kit v2; Thermo Fisher Scientific, Italy) of the first and last swab (Fig. 1).

As shown in the amplification plot in Fig. 1, in the first swab (a) all three tested genes were positive, with cycle threshold (Ct) values of 33 (S gene), 32 (N gene), and 31 (ORF1ab gene). The amplification plot of the swab on day 41 (Fig. 1b) showed clear positivity of two of the three tested genes (S gene: Ct 37 and ORF1ab gene: Ct 36) and a doubtful result of the N gene (Ct 39). Nonetheless, the last swab was reported as positive due to the positivity of two of the three genes tested, according to the TaqMan 2019-nCoV Assay Kit v2 indications.

**Fig. 2:**
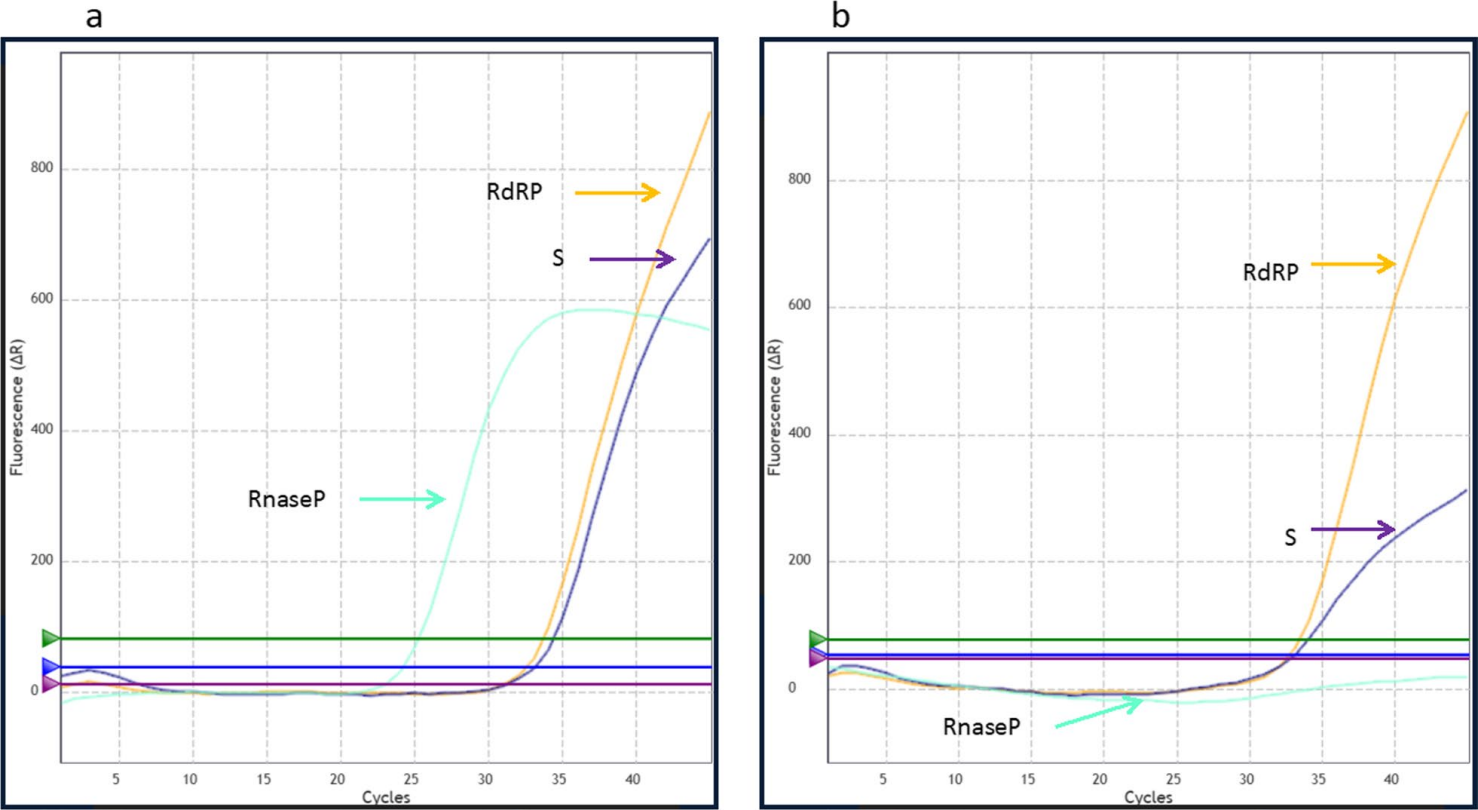
RT-PCR (Realquality RQ-SARS-CoV-2, AB Analytical) analysis results. First swab **a** showed positivity for both viral (S and RdRP genes, identified by purple and yellow arrow, respectively) and cellular (RnaseP, identified by the green arrow) genes. Day 41 swab **b** showed positivity for the two viral genes, while the cellular gene was not detectable

To confirm the positivity and to obtain a cellularity check on the swabs, we then analyzed the samples with another RT-PCR kit (Realquality RQ-SARS-CoV-2, AB Analytical) (Fig. 2).

Interestingly, we found that the first swab showed positivity for both viral (S and RdRP genes) and cellular (RnaseP) genes (a). Conversely, after 41 days, the two viral genes were still positive, while the cellular gene was not detectable (b)

It was not possible to perform other swabs beyond 41 days due to burial authorization and also the putrefactive state of the body (batrachian abdomen with abdominal and facial putrefactive spottings).

## Discussion and conclusions

The pandemic situation has led to an intensification of scientific research to provide new data on SARS-CoV-2 virus behavior. Since the beginning of the worldwide spread of SARS-CoV-2 to date, important knowledge has been obtained about the virus behavior in living subjects and on inanimate surfaces [8–13]. Van Dormagen *et al.* [14] detected SARS-CoV-2 on surfaces after 5 days at a temperature between 22 °C and 25 °C and 40–50% humidity, as well as on plastic after 72 hours, on stainless steel after 48 hours, and on copper after 8 hours. Another study conducted by Moriarty [15] on a cruise ship reported that SARS-CoV-2 RNA could be detected in the cabins up to 17 days after the passengers got off the ship. However, there is a lack of data on virus persistency on dead bodies and on the risk of contagion from cadavers. These data are of fundamental importance as the execution of the autopsies puts various categories of workers (pathologists, technicians, and so on) in contact with a possible source of biological risk [16, 17], with implications not only from a health point of view but also from a medicolegal one.

Specific guidelines have been developed to allow the execution of safe postmortem investigations [18–21], considering the available data. To date, we could not find any recent publication nor WHO report defining the risk of postmortem transmission of SARS-CoV-2 or how long body fluids remain infected in the cadaver.

In a study by Dijkhuizen *et al.* [22], they tested viral RNA postmortem via nasopharyngeal and oropharyngeal swabs and detected it up to 27 hours after death. The longest reported persistence of SARS-CoV-2 RNA on a cadaver is 35 days [23]. However, neither of these studies were able to define the contagiousness of the virus.

The present case shows the persistence of SARS-CoV-2 RNA up to 41 days after death. Data about its persistence are of fundamental importance for pathologists to understand when the handling of the corpse is safe, and further studies are needed to estimate the virus contagiousness. Claims have also been reported about possible infection from a corpse for a few hours up to a day after death, but these statements are not supported by scientific data *in vivo*. Nonetheless, a study conducted by Heinrich *et al.* [24] shows maintained infectivity of a corpse; they took a tissue sample from the throat of SARS-CoV-2 deceased patients and found that the virus could replicate up to 35 hours postmortem.

The main route of virus transmission is inhalation of large respiratory droplets, but transmission via contact with contaminated body excretions, air, and fecal–oral route has also been suggested. When managing SARS-CoV-2-positive cadavers, the potential risk of transmission may be related to direct contact with human remains or body fluids where the virus is present, or to direct contact with contaminated fomites. Due to the postmortem presence of the virus, it is advisable to minimize the contact and exposure to infected body fluids, paying particular attention to the procedures that generate aerosols during an autopsy [25]. Although an analysis of 75,464 SARS-CoV-2 cases in China [26] did not report any transmission other than via droplets, airborne transmission may be possible in specific circumstances in which procedures or support treatment are performed that generate aerosols. Currently, no evidence of virus transmission due to manipulation of infected corpses has emerged. One case seems to have been reported, even if the authors do not scientifically confirm that the infection occurred from a corpse [27, 28]. Another study conducted by Han *et al.* [29] also reported no disease symptoms in autopsy participants after 24 examinations on SARS-CoV-2-positive bodies. Therefore, there is currently no scientific evidence proving contagiousness from a SARS-CoV-2-positive corpse [22, 30, 31]. This case may contribute to increase the knowledge on SARS-CoV-2 persistency in dead bodies.

This case shows a long resistance of the viral genome in a corpse kept in controlled environmental conditions (cold room temperature 4 °C) 41 days after death, with a low initial viral load. The man, who died from drowning, was completely asymptomatic when he was alive. Our data reflect the importance of postmortem swabs in all autopsy cases because asymptomaticity does not exclude the presence of the virus. Moreover, we found that only the viral genes, but not the cellular RNA, persisted after 41 days.

We are aware of the limitations of our study, mainly related to the exiguity of the sample, and further studies are needed to confirm postmortem survival of the virus and assess the possible risk of postmortem contagiousness.

## Data Availability

Not applicable.

## Abbreviations

SARS-CoV-2: Severe acute respiratory syndrome coronavirus 2
RT-PCR: Real-time reverse transcriptase-polymerase chain reaction
